# Interventions to Reduce Serum Per- and Poly-Fluoroalkyl Substances Levels, Improve Cardiovascular Risk Profiles, and Improve Epigenetic Age Acceleration in US Firefighters: Protocol for Randomized Controlled Trial

**DOI:** 10.2196/67120

**Published:** 2025-04-16

**Authors:** Reagan Conner, Cynthia Porter, Karen Lutrick, Shawn C Beitel, James Hollister, Olivia Healy, Krystal J Kern, Floris Wardenaar, John J Gulotta, Kepra Jack, Matthew Huentelman, Jefferey L Burgess, Melissa Furlong

**Affiliations:** 1 Department of Community, Environment and Policy Mel and Enid Zuckerman College of Public Health University of Arizona Tucson, AZ United States; 2 Department of Epidemiology and Biostatistics Mel and Enid Zuckerman College of Public Health University of Arizona Tucson, AZ United States; 3 Family and Community Medicine College of Medicine - Tucson University of Arizona Tucson, AZ United States; 4 College of Health Solutions Arizona State University Phoenix, AZ United States; 5 Tucson Fire Department Tucson, AZ United States; 6 HeartFit For Duty Mesa, AZ United States; 7 Neurogenomics Division The Translational Genomics Research Institute Phoenix, AZ United States

**Keywords:** firefighters, PFAS, epigenetics, phenotype, heart disease, cardiovascular disease, CVD, atherosclerosis, occupational health, RCT, cardiovascular, fasting, exercise

## Abstract

**Background:**

Occupational cancer and acute cardiac events are the leading causes of death among firefighters. Increased exposure to toxicants on the fire ground, such as polycyclic aromatic hydrocarbons, benzene, and per- and poly-fluoroalkyl substances (PFAS), has been linked to certain cancers, cardiovascular disease, accelerated epigenetic aging, and other adverse health effects. PFAS are a major concern because they are persistent, can bioaccumulate, and are present in several firefighting tools. Compared to the general population, firefighters have elevated serum levels of some types of PFAS. A randomized clinical trial in Australian firefighters found that routine blood and plasma donation for 1 year led to decreased serum PFAS levels, although health outcomes were not directly measured in that study.

**Objective:**

In collaboration with fire service leadership in Arizona, the Firefighter Collaborative Research Project (FCRP) was established to evaluate the effectiveness of 3 interventions in a randomized controlled trial design to reduce serum PFAS levels, reduce cancer and cardiovascular risk, and improve overall health and wellness in US firefighters.

**Methods:**

This study aimed to recruit and enroll up to 1500 active firefighters between August 2023 and October 2024. Between August 2023 and October 2024, active firefighters were recruited and randomized into a study arm based on their eligibility, including serum PFOS levels, for the specific arms. The trial arms include (1) blood and plasma donation, (2) zone 2 physical activity, and (3) intermittent fasting. FCRP outcomes include serum PFAS reduction (arm 1), epigenetic age acceleration (all arms), cardiovascular conditioning (arm 2) and cognitive outcomes (all arms), mental health (all arms), and overall disease risk (all arms). Each study arm includes an intervention and a control group. At enrollment and end of the study, participants provide blood and urine samples and complete a comprehensive questionnaire on their occupational and health history, exposures, and lifestyle behaviors. At the end of the study, participants also participated in a cognitive evaluation. Depending on the study arm, participants may additionally complete a cardiopulmonary exercise test at baseline and follow-up, a mid-study survey, and a mid-study blood and urine collection.

**Results:**

Participant activities and data collection will conclude by December 2025.

**Conclusions:**

The FCRP is a randomized controlled trial that aims to test the effectiveness of fire service–selected interventions in reducing serum PFAS levels. Study results will contribute to potential interventions that could be used to reduce serum PFAS levels in firefighters.

**Trial Registration:**

ClinicalTrials.gov NCT05869747; https://clinicaltrials.gov/study/NCT05869747

**International Registered Report Identifier (IRRID):**

DERR1-10.2196/67120

## Introduction

Occupational cancer and acute cardiac events are the leading causes of death among firefighters [1,2]. The firefighting occupation is classified by the International Agency for Research on Cancer as a group 1 carcinogen due to the direct and indirect exposures to hazards on the job [3]. Firefighters have a significantly higher risk of cancer and cancer mortality when compared to the general population, including skin melanoma, other skin cancers, and prostate cancer, with higher mortality for rectal, testicular, brain and nervous system cancers, and non-Hodgkin lymphoma [4,5]. Increased exposure to toxicants on the fire ground, such as polycyclic aromatic hydrocarbons, benzene, and per- and poly-fluoroalkyl substances (PFAS), have been linked to certain cancers, cardiovascular disease, accelerated epigenetic aging, and other adverse health effects [4,6,7].

PFAS are primary toxicants of concern due to their persistence, ability to bioaccumulate, and associated adverse health effects [8]. Firefighters have additional exposure to these chemicals beyond the general population, with significant sources of PFAS exposure on the fire ground including fluorinated class B aqueous film-forming foam and smoke from burning household materials [6]. A study involving 4 municipal US fire departments located in different states and regions of the country found that firefighters had higher levels of several PFAS chemicals in their blood than the general population [9].

In the human body, PFAS accumulates primarily in the liver, kidneys, and blood. Long-chain PFAS, such as perfluoro octane sulfonate (PFOS) and perfluorooctanoic acid (PFOA), have reported half-lives of several years (3.1-7.4 and 2.1-8.5, respectively) [8]. These chemicals bind strongly to albumin, the most abundant protein in plasma [8,10]. Therefore, these chemicals may be removed from the body through blood or plasma donation. An Australian study among firefighters previously demonstrated the effectiveness of blood or plasma donation to lower serum PFAS levels over 12 months, although no health effects were investigated. Findings from the study revealed that plasma donation was more effective than blood donation at reducing the mean serum level of PFOS at 12 months, while serum PFOS did not significantly change in the control group [11]. Plasma donation reduced PFOS levels by approximately 24% (–2.9 ng/mL, 95% CI –3.6 to –2.3 ng/mL), while blood donation reduced PFOS levels by approximately 11% (–1.1 ng/mL, 95% CI –1.5 to –0.7 ng/mL) [11]. Based on the findings from that study and concern for PFAS exposure, Arizona firefighters requested research to replicate the study and additionally investigate health outcomes such as cardiovascular health and epigenetic age acceleration.

Lifestyle and environmental exposures are associated with DNA methylation patterns, a type of epigenetic alteration. DNA methylation affects gene expression by adding a methyl group to 5’—C—phosphate—G—3’ dinucleotides. These DNA methylation patterns can be analyzed to determine epigenetic age or “biological age” through epigenetic clocks. Previous research has found that accelerated epigenetic age can be a risk factor for cancer, cardiovascular and neurological diseases, as well as death from all causes combined [12]. In addition to the physical health impacts, accelerated epigenetic age has been linked to negative mental health impacts [13]. Studies on firefighters have found differential DNA methylation patterns, or accelerated epigenetic age, potentially as a result of fire ground exposure and increased serum PFAS levels [7,14].

Lifestyle interventions, such as physical activity and dietary restriction or intermittent fasting, may be associated with slowed epigenetic aging [12,15,16]. Findings from the Comprehensive Assessment of the Long-term Effects of Reducing Intake of Energy trial revealed that caloric restriction slowed epigenetic aging [17]. Similar findings have been observed in mice [18]. A recent randomized controlled trial tested the effectiveness of a fasting-mimicking diet (low calorie, low protein, plant-based diet) in improving biological age. After 3 cycles, a cycle being a consecutive 5-day period in a month, biological age was reduced by a median of 2.5 years [19].

Zone 2 physical activity, or continuous, moderate aerobic physical activity corresponding to the near lactate threshold (4 mM blood lactate), shows greater benefits than other types of aerobic physical activity for improving cardiovascular risk factors [20,21]. Common examples of this type of activity include brisk walks, light jogs, and cycling for a continuous interval of time. Occupational health clinicians working with the firefighter community also anecdotally noted that zone 2 physical activity confers the greatest benefits for cardiovascular fitness, although this claim has not been formally tested. Both types of lifestyle interventions, intermittent fasting, and zone 2 physical activity, have received interest from the firefighter community.

The Firefighter Collaborative Research Project (FCRP) aims to assess the effectiveness of fire service–selected interventions for improving firefighter health. The target population for recruitment is participants enrolled in the Fire Fighter Cancer Cohort Study (FFCCS), a national framework study that collects information on firefighter exposures and works to improve the health of the fire service [22]. Through this randomized controlled trial, we seek to engage our existing cohort of firefighters to evaluate 3 firefighter-identified lifestyle interventions: blood or plasma donation, zone 2 physical activity, and intermittent fasting. The primary outcomes of the study include epigenetic age acceleration, serum PFAS reduction (blood or plasma arm only), and cardiovascular risk reduction (zone 2 arm only). Based on the previously mentioned associations with accelerated epigenetic age and therefore aging and aging-related diseases, secondary outcomes of this study include mental health, sleep quality, and cognitive functioning [12,13].

## Methods

### Overall Study Design

The FCRP is a community-engaged, randomized controlled trial with the goal of screening 1500 firefighters. The study enrollment period is planned for August 2023-October 2024. Based on eligibility criteria, participants are randomly assigned to regularly donate blood or plasma for 1 year, engage in zone 2 physical activity for 4 months, intermittent fast for 4 months, or serve as a control for the duration of the respective study arm.

We have chosen a community-based approach working with fire service leaders in participating states that are willing to educate firefighters on the importance of the study and encourage participation. Departments designate a liaison who completes training for institutional review board (IRB) approval to distribute study information, coordinate events, and serve as an additional point of contact between researchers and participants. The study team also hosts online informational sessions upon request and actively maintains a study website. Individuals interested in participating in the study are directed to complete a screening survey and to schedule an appointment for an in-person event near them. Both surveys may be accessed through informational materials dispersed by liaisons. Events are hosted regionally, and locations are prioritized based on interest numbers and resources for study activities.

Initially, the state of Arizona was designated as the only state for enrollment for all 3 study arms (blood and plasma donation, intermittent fasting, and zone 2). However, upon interest from other US fire departments, the blood and plasma donation arm of the trial has been expanded to Idaho, California, Washington, Oregon, and Massachusetts. The intermittent fasting and zone 2 arms of the trial will not include non-Arizona fire departments. Given that there are a limited number of volunteer departments with who we have established contacts within these areas, enrollment is focused on career departments. Firefighters of all demographic backgrounds were encouraged to enroll.

### Participants

#### Eligibility Criteria

Individuals interested in participating in the study are directed to complete an online screening survey to assess eligibility. Overall study eligibility criteria include active firefighters enrolled in the FFCCS, with full criteria described in Textbox 1. If participants are not already enrolled in the FFCCS at recruitment, they are given the opportunity to enroll. Individuals currently doing study arm activities at the time of screening are deemed ineligible for participation in the respective study arm. Subjects with conditions that may affect their ability to complete study activities (eg, they do not meet national standard criteria for blood or plasma donation, or have an injury preventing physical activity) are also deemed ineligible for participation in the respective study arm. Individuals who are deemed ineligible for all study arms are thereby ineligible for participation in the overall study. Specific inclusion criteria are detailed in Textbox 1.

**Inclusion and exclusion criteria for the Firefighter Collaborative Research Study (FCRP).**

**Inclusion Criteria**

- Active firefighter (including emergency medical responder and all firefighter subgroups) with either a volunteer or career status
- Individual plans to remain in active service with their current agency for the next 2 years (not planning to retire or resign)
- 18 years of age and older
- Fluently speak and write in English
- All genders, races, and ethnicities
- Enrolled in the Fire Fighter Cancer Cohort Study before enrolling in the FCRP
- Complete a signed and dated informed consent document that indicates the participant has been informed of all aspects of the study before enrollment.
- Agree to avoid participating in FCRP intervention activities outside of their assigned intervention group for the duration of the study.
- Able to comply with scheduled visits, laboratory tests, and other study procedures
- BMI ≥17.5 kg/m^2^ and weighing more than 115 pounds

**Exclusion Criteria**

- Overall
  Not able to fluently speak or write in English
  Younger than 18 years
  Currently a tobacco smoker or vaping (eg, >2 cigarettes or cigars, or incidents of vaping in the past month)
  Those with planned travel or extended leave (eg, >6 weeks) that would prevent their ability to participate in other interventions
  Those who are pregnant or become pregnant during study, breastfeeding, or have given birth within the past year
  Those with a history or diagnosis of any significant metabolic, hematologic, pulmonary, cardiovascular, gastrointestinal, neurological, immune, hepatic, renal, urological disorders, severe injury, or cancer that, in the opinion of the investigator, would potentially put the candidate at risk.
- Blood or plasma
  Those with any medical contraindication (medical condition or medication) to blood donation, according to Red Cross Guidelines
  Those who donated blood or plasma in the past 3 months
  Those knowing they have a condition indicative of levels of hemoglobin, hematocrit, red blood cells, or iron below the lower limit of normal levels
- Zone 2
  Currently participating in zone 2 physical activity
  Currently participating in more than 120 minutes per week of aerobic, and cardiovascular training (eg, jogging, cycling, walking, swimming, high-intensity interval training) at >60% of their max heart rate.
- Intermittent fasting
  Those with a history or diagnosis of diabetes, hypoglycemia thyroid disease, or an eating disorder
  Those who recently participated in intermittent fasting or Time Restricted Eating
  Those who recently used antidiabetic medication such as Semaglutide (sold as Ozempic, Wegovy, and Rybelsus) or Tirzepatide (sold as Mounjaro) for the treatment of type 2 diabetes or weight loss will also be excluded from this group.
  Use of drugs that might affect intermittent fasting or eating behaviors

#### Interventions

Randomization groups are created at regular monthly intervals and clustered by recruitment region. Participants who have consented to participate in the study are randomized into 1 of the 4 study arms as either an intervention or control, accounting for their eligibility determined during the screening process and baseline serum PFOS concentrations. Upon enrollment into their specific group, participants are provided with instructional materials for assigned activities, activity timelines, and frequently asked questions.

Participants randomized in the blood donation or plasma donation arm include all participants with serum PFOS ≥5 ng/mL, following the previous Australian study [11], while also meeting additional study criteria outlined in Textbox 1. We also included a smaller intervention group of participants with PFOS <5 ng/mL in the plasma arm, to evaluate whether improvements in epigenetic age are independent of baseline PFOS. Participants in the blood intervention arm are expected to donate whole blood every 12 weeks for 12 months, while those in the plasma intervention are expected to donate plasma every 6 weeks for 12 months. Participants are provided with a list of donation centers in their region and acceptable types of donations (eg, whole blood donation is acceptable, but not “Power Red” or platelets). Participants go to donation centers on their own. Controls are asked to refrain from donating plasma or blood throughout the trial period.

Participants randomized into the zone 2 physical activity arm complete a Cardiopulmonary Exercise Test (CPET) before starting their intervention. The CPET uses a mouthpiece and external sensors to measure the heart rate and other cardiovascular health indicators of the individual while they engage in mild exercise on a stationary bicycle. The initial CPET provides a baseline evaluation of cardiovascular fitness, as well as the zone 2 heart rate zone used for the intervention. The calculated range from this exam is provided to participants in their results and they are asked to self-report these results to the study team. The zone 2 heart rate range was based on current American College of Sports Medicine guidelines and corresponded to a lower limit of 40% and an upper limit of 60% of achieved peak VO2 to indicate the anaerobic threshold [23]. Functionally, engaging in zone 2 translates to moderate physical activity, with examples of zone 2 physical activity including hiking up a hill, a light jog, or a moderate cycling session. Participants in the intervention are asked to complete a minimum of 45 minutes of physical activity in their zone 2 range for at least 4 days a week, tracked by a study-provided heart rate monitor watch. Controls are permitted to continue their regular physical activity regimen but are asked to not intentionally engage in zone 2 activity. All participants randomized to the zone 2 physical activity arm are provided with a wrist-worn accelerometer (Garmin Forerunner 45) to passively record their physical activity and sleep data. They are reminded to always wear the device except when it is charging and receive regular compliance checkups from study staff. Participants are also reminded to open the application on their phones at least once a week to allow data to be uploaded to their Garmin account. This data is synced with Fitabase to allow the study team to monitor adherence. Participants in the zone 2 physical activity arm are monitored for compliance through Fitabase and can manually report exercises if they do not wear their wrist-worn heart tracker [24]. At the end of the 4-month period, participants complete a final CPET exam and share their results with the study team to evaluate changes over time.

Participants randomized into the intermittent fasting arm are asked to fast for 14 to 16 hours per day, for at least 4 days a week for the 4-month period. Participants select their own time windows to accommodate firefighter shift schedules. During fasting windows, participants are allowed to consume medications and zero-calorie beverages (eg, black coffee, sparkling water, unsweetened tea). They are not asked to alter their diet in any other manner. Controls are asked to not engage in intermittent fasting throughout the trial period and continue their current dietary regimen. Additional details are provided in Textbox 2.

**Description of study interventions within the firefighter collaborative research project.**

**Arm 1: Blood or plasma donation**

- Blood or plasma donation
  Participants with perfluorooctane sulfonate >5 ng/mL were prioritized for this arm
  Blood: 12-month commitment to donating blood every 12 weeks. Each blood donation will take approximately an hour to an hour and 15 minutes each time.
  Plasma: 12-month commitment to donating plasma every 6 weeks. Each plasma donation will take approximately an hour and 30 minutes to 2 hours each time.
  Additional surveys administered will involve a time commitment between 30 to 45 minutes.
- Blood or plasma control group
  12-month commitment into the control group
  Additional surveys administered will involve a time commitment between 30 to 45 minutes.

**Arm 2: Zone 2 physical activity**

- Zone 2 physical activity intervention
  4-month commitment to completing a minimum of 45 minutes of physical activity in zone 2 for a minimum of 4 days per week.
Cardiopulmonary Exercise Test (CPET) testing before beginning and after completing the zone 2 arm for 4 months.
  4-month commitment to wear a wrist-worn heart and health tracker at all times unless it is charging.
  Additional surveys administered will involve a time commitment between 30 to 45 minutes.
- Zone 2 control group
  4-month commitment to wear a wrist-worn heart and health tracker at all times unless it is charging
  CPET testing before beginning and after completing the participation in zone 2 control group arm for 4 months.
  Additional surveys administered will involve a time commitment between 30 to 45 minutes.

**Arm 3: Intermittent fasting**

- Intermittent fasting intervention
  4-month commitment. The participant will limit intake of food and calorie-containing beverages to an 8-10 hour window a day and fast for the remaining 14-16 hours on 4 days per week.
  Additional surveys administered will involve a time commitment between 30 to 45 minutes.
- Intermittent fasting control group
  4 month-commitment into the control group
  Additional surveys administered will involve a time commitment between 30 to 45 minutes.

#### Data Management

Research activities primarily occur through electronic communications such as email, text, and internet-based surveys, telephone contacts, or postal or express mail. Surveys are self-administered by participants on a computer or smartphone. Should participants be unable or unwilling to access them online, they may be administered by study staff by telephone. Participant information given to study staff via phone or email conversation is entered and stored in the REDCap database [25].

The database for this study is maintained through REDCap, which securely keeps participant identifiers and contact information according to the University of Arizona’s standard operating procedures. These procedures operate with respect to cybersecurity, privacy, patient confidentiality, and compliance with applicable patient privacy regulations. All study-related documents and specimens contain a unique identifier for each participant, with any study-related documents with personal identifiers stored in a locked cabinet in lockable offices on campus. Laboratory results are entered directly into the REDCap study database from the study reference laboratory.

Twilio is a cloud-based communications platform that allows for automated text messaging chains to be sent to study participants. It is used to send study survey links to their phones that will take them directly to secure REDCap surveys.

At enrollment and their end-of-study visit, all participants provide biological specimens (blood and urine), and a survey is administered to collect information on occupational history and exposures, relevant medical history, sleep quality, physical activity information, a food frequency questionnaire, the Center for Epidemiological Studies Depression scale (short form), and environmental exposures (Table 1, and described further in the Outcomes section). Throughout their enrollment period, all participants also receive regular study adherence questionnaires (described below). At end-of-study only, all participants additionally take a cognitive assessment (MindCrowd, described below). Participants assigned to the zone 2 physical activity arm complete a CPET before the beginning of the 4-month intervention and again after the conclusion of the intervention. Once participants in the zone 2 physical activity arm report the results of their first CPET, they are sent a wrist-worn accelerometer (Garmin Forerunner 45) that they are instructed to wear throughout the 4-month period and to return upon completion.

Zone 2 wears their accelerometer every day for the duration of the trial.

**Table 1:** Participant data collection time points for participants enrolled in the firefighter collaborative research project.

	Baseline	Mid-intervention	End of study^a^
Blood	All	BP^b^	All
Urine	All	BP	All
Questionnaires^c^	All	BP	All
Adherence^d^	N/A^e^	All^f^	N/A
Cardiopulmonary Exercise Test	Z2^g^	N/A	Z2
MindCrowd cognitive assessment (verbal cognition, processing speed)	N/A	N/A	All
Accelerometer	Z2	Z2	Z2
Epigenetics (Infinium MethylationEPIC v2.0 BeadChip)	All	N/A	All
VO2max, resting heart rate, HRV^h^, HR^i^ recovery	Z2	Z2	Z2
Sleep quality (accelerometer): deep, REM^j^, light	Z2	Z2	Z2
Sleep quality (National Health Interview Survey Questionnaire): hours, rested, interruptions	All	N/A	All
Depression (CES-D^k^ Short form)	All	N/A	All
Firefighter gear and occupational exposures	All	N/A	All
Dietary quality	All	N/A	All
Self-reported weight	All	All	All

^a^End of the study is approximately 4 months after randomization for zone 2 and intermittent fasting and approximately one year for blood or plasma.

^b^BP: blood/plasma arm.

^c^Questionnaires include diet, depression or mood, physical activity, occupational characteristics, and lifestyle.

^d^Intervention adherence questionnaires are evaluated weekly for IF and Z2, and monthly for blood or plasma.

^e^N/A: not applicable.

^f^All: all arms.

^g^Z2: Zone 2 physical activity.

^h^HRV: heart rate variability.

^i^HR: heart rate.

^j^REM: rapid eye movement.

^k^CES-D: Center for Epidemiologic Studies Depression Scale.

#### Biological Specimens

All participants provide up to 50 mL of blood via venipuncture at baseline as part of the study screening process and at the completion of assigned study arm activities. Participants in the blood and plasma arm complete an additional collection at mid-study, between 4 and 8 months. Each venipuncture is performed by qualified study staff, phlebotomists, nurses, or paramedic-EMTs at organized study events hosted at participating fire stations or facilities. Upon collection, blood specimens are stored locally in coolers with ice packs for transportation to the University of Arizona, where they are processed within 24 hours, with aliquots stored at –80 °C before testing. Baseline blood specimens for all participants are analyzed for PFAS as the final step in the study screening process and for markers of epigenetic age, such as DNA methylation patterns. At the end of assigned activities, only participants in the blood and plasma arm will have their blood specimens analyzed for PFAS. All blood specimens collected at the end of assigned activities for all study arms will be analyzed for markers of epigenetic age.

Participants are asked to self-collect approximately 80 mL of urine at baseline as part of the study screening process and at the completion of assigned study arm activities per FFCCS protocol and to be stored for future analyses. Participants in the blood and plasma arms complete an additional collection at mid-study, between 4 and 8 months. Collections take place at organized study events hosted at participating fire stations or facilities. Upon collection, urine specimens are locally stored on dry ice for transportation to the University of Arizona, where specific gravity is measured using the Atago Refractometer (Model PAL-10S, Cat# 4410, Fisher Scientific). The sample is aliquoted and prepared for long-term storage at –80 °C for potential future analyses associated with these interventions.

#### Adherence to Study Activities and Retention

To determine activity adherence, participants are contacted at regular intervals determined by their intervention via email or secure SMS test messages using Twilio. Each survey asks if the participant has completed their required study activities. Participants randomized to an intervention are asked detailed questions about completing their activities, and participants randomized to control are asked if they engaged in the intervention activities since the last study survey. During the check-in surveys, participants are able to report any adverse events. They will then be contacted by study coordinators, and the events will be reported to the IRB for review to determine any necessary actions to take.

Study coordinators call and email noncompliant participants each week to improve adherence. Participants are contacted if they are due to start study activities but have not completed their baseline survey, have at least 1 week past due for a blood or plasma donation, have not completed a weekly survey for zone 2 physical activity or intermittent fasting in 2 weeks, or have not scheduled an appointment for a follow-up biological collection. Participants may also be contacted if they do not sync their wrist-worn accelerometer (Garmin Forerunner 45) with their mobile application, or if they do not share the results of their CPET with the study team. If the participant does not respond and continues to fall behind on study activities, then the IRB-approved liaison for their department is contacted to engage with the participant and ensure they complete their activities.

### Laboratory Methods

#### Per- and Poly-Fluoroalkyl Substances Concentrations

Serum samples are tested for PFAS by the New Jersey Department of Health Centers for Disease Control method #6304.09 as a reference using Chronos online SPE – uHPLC by Spark Holland coupled with a Sciex 7500 MS/MS. A total of 20 analytes are tested, including perfluorobutanesulfonic acid, perfluorodecanoic acid, perfluoroheptanesulfonic acid, perfluoroheptanoic acid, perfluorohexanesulfonic acid, perfluorohexanoic acid, perfluorononanoic acid, perfluorooctanesulfonamide, perfluoroundecanoic acid, PFOA (perfluorooctanoic acid), PFOS, branched and linear isomers of PFOA and PFOS, 9-Chlorohexadecafluoro-3-oxanonane- 1-sulfonic acid, 4,8-Dioxa-3H-perfluorononanoic acid, 2-(N-Ethyl-perfluorooctane sulfonamido) acetic acid, 2-(N-Methyl-perfluorooctane sulfonamido acetic acid), and Hexafluoropropylene oxide dimer acid. Note that total PFOA and total PFOS are calculated as the sum of the branched and linear isomers [26].

#### DNA Methylation Analysis

DNA methylation analysis of serum samples is conducted at the University of Arizona Genetics Core. After sample accessioning and preparation, genomic DNA is extracted using a Qiagen DNeasy Blood and Tissue Kit (REF: 69581) or similar following the manufacturer’s recommendations for whole blood. The extracted DNA is then quantified using promega quantiflor fluorometric dsDNA assay (PN: E2671) and dsDNA quantities are detected using a SynergyHT plate reader (Agilent or Biotek). Samples are then normalized or diluted robotically with HPLC water to the range of 200-500ng in a total volume of 45uL. Following normalization, a bisulfite conversion using the Zymo EZ-96 DNA Methylation Kit (PN: D5004) is performed with the specified thermocycler conditions (95 °C for 30 sec, 50 °C for 60 min for 16 cycles, and Hold” at 4 °C) with 15uL as the final elution volume.

The bisulfite-converted DNA is then input into the illumina infinium methylation EPIC Kit (PM 20087706-8) following the manufacturer’s recommendations. Whole genome amplification is then performed on the bisulfite-converted DNA. The amplified DNA is then fragmented and precipitated before being resuspended and hybridized onto the bead chip (v2.0) during an overnight incubation. Unhybridized DNA is washed off before the bead chip undergoes single base extension and staining. Before scanning, the bead chip undergoes a final round of washing and coating to prepare the chip for imaging. Once prepped for imaging, the bead chip is then scanned on an Illumina NextSeq 550 following the manufacturer’s recommendations.

### Outcomes

The primary outcomes of the study include epigenetic age acceleration, serum PFAS reduction, and cardiovascular risk reduction, with secondary outcomes including mental health, sleep quality, and cognitive functioning. Intervention-specific outcomes include PFAS concentrations (plasma or blood arm), and cardiovascular fitness (zone 2 arm). Urine and blood samples are stored at –80 °C for future biomarker-based outcomes. In total, 20 PFAS types are measured for all participants at baseline and again at end-of-study for participants in the blood and plasma study arm. Participants are provided with a report back of their PFAS concentrations at baseline and again at follow-up if measured. Epigenetic age acceleration will be calculated for baseline and end-of-study samples for all participants using several different clock-based methods, including the Horvath clock, GrimAge, Hannum, SkinBlood, PhenoAge, and Dunedin PACE [26-28]. Epigenetic age acceleration will be calculated using residuals after regressing each clock on chronological age [29,30]. Participants who complete activities required by the study protocol will have their epigenetic age acceleration reported back to them.

Cognitive functioning is measured with the MindCrowd web-based platform created by TGen. MindCrowd measures verbal memory performance using paired associates learning, and processing speed via simple visual reaction time. Both tests are strongly predictive of age and functional ability across the lifespan, and MindCrowd has been validated in hundreds of thousands of participants across the world [31-33]. To minimize the time burden on enrollment and possible learning effects, MindCrowd is only assessed at follow-up.

Cardiovascular fitness is evaluated with baseline and end-of-study CPETs, and additionally on an ongoing basis with wrist-worn accelerometers. The CPETs produce validated measures of resting VO2 (maximal aerobic capacity), peak VO2, VO2 reserve, and heart rate. The accelerometers from the wrist-worn watch also generate an estimate for peak VO2, and we will assess the progression of this over time as a function of intervention status.

Depression and mood are evaluated at baseline and follow-up using the revised version of the Center for Epidemiologic Studies Depression Scale. Diet quality is measured from a food frequency questionnaire at baseline and follow-up. Sleep quality is measured via the wrist-worn accelerometers for those in the zone 2 arm, and through a modified version of the Centers for Disease Control’s National Health Interview Survey’s Sleep Surveillance questionnaire for all participants. The National Health Interview Survey sleep survey is slightly modified to account for firefighter’s shift work.

### Statistical Considerations

#### Power Analysis

We conducted a power analysis based on the outcomes of changes in PFAS concentrations, as well as changes in epigenetic ages. Following the initial Australian study, to see a change in concentration of 20% PFOS from before and after, we calculated an N of approximately 100 participants per group or 300 total for the blood, plasma, or control groups. However, to observe a change of one year of epigenetic age acceleration, with a standard deviation of 3, we required an N of 141 per group. To account for missingness, we are targeting an N of 150 per group.

Therefore, the targeted sample size for PFOS ≥5 ng/mL is 150 plasma test participants, 150 blood test participants, and 150 shared controls for blood or plasma; and for PFOS <5 ng/mL we are targeting 75 intervention participants; for zone 2 is 150 controls and 150 test participants; for intermittent fasting, sample size is 150 controls and 150 test participants. Thus, the total number of participants that complete the study is targeted at 1120. Randomization of the intervention groups is conducted using R v4.2.0 [34].

#### Data Analysis

All models will evaluate intent to treat effects, as well as the average treatment effect of the treatment. We will first evaluate characteristics by treatment group to ensure randomization is effective.

To estimate the effect of blood and plasma donation on the reduction of PFAS concentration, mixed effects models with random effects by department or region will be fit to compare changes in PFAS concentration before and after the study among intervention groups. We will report mean differences in the change from baseline to end-of-study measurements by group and adjust for residual confounders. We will also evaluate whether the volume of plasma and blood donated is a significant predictor of PFAS change, and account for adherence in these models. The results of these models will evaluate if there is a statistically significant difference in the change of PFAS concentration from baseline to follow-up, in cases compared to controls, and if there is a dose-response relationship between volume donated and PFAS concentration change.

Linear mixed-effect models will also be used to examine differences between groups in epigenetic age acceleration, cardiovascular fitness, cognitive functioning (measured only at follow-up), mental health, sleep quality, and other measured outcomes. Similar models will be used for zone 2 physical activity and intermittent fasting interventions. Missing data will be assessed and if substantial missing data is identified (greater than 10%), a sensitivity analysis will be conducted using multiple imputations by chained equations.

### Ethical Considerations

This study was reviewed and approved by the University of Arizona IRB (STUDY00002462). All participants complete informed consent electronically through the REDCap database system and all collected identifiable data is stored within the database (Multimedia Appendix 1). The research staff verifies that participants fully understand all risks and the necessary study activities that are required of them. Once participants agree to be active participants in the study, the study staff sign the informed consent. All participants receive the results of their PFAS concentrations, measured from the baseline blood collection during the screening process. No monetary compensation is provided to any participant in the study. At the end of study activities, only participants in the blood and plasma donation arm will receive a second set of PFAS results, measured from the blood collection at the end of the study. In addition, following the completion of all study activities, all participants receive the results of their epigenetic clocks.

Any participant information provided in this manuscript is de-identified. Any identifiable information is stored within the REDCap database, which securely keeps participant identifiers and contact information according to the University of Arizona's standard operation procedures. These procedures operate with respect to cybersecurity, privacy, patient confidentiality, and compliance with applicable patient privacy regulations. All study-related documents and specimens contain a unique identifier for each participant, with any study-related documents with personal identifiers stored in a locked cabinet in lockable offices on campus.

## Results

This study was funded by the Arizona Board of Regents. Enrollment began in August 2023 and concluded in November 2024. As of January 2025, a total of 916 participants were enrolled in the study and 1893 participants were screened. The conclusion of the blood and plasma interventions is set for December 2025, contingent upon funding. Data analysis will begin once sufficient data has been collected.

## Discussion

### Overview

All study interventions were selected based on interest from a community-engaged participatory framework with the fire service. Testing the effectiveness of blood and plasma donation in reducing serum PFAS levels in firefighters is a high priority among firefighters, given that this population has been found to have higher serum levels of these chemicals compared to the general population [6,9]. Evaluating all interventions as potential ways to improve epigenetic age acceleration and reduce disease risk is a priority in improving the health of the fire service. A primary strategy for promoting participant adherence to study activities is by reporting the results of their serum PFAS concentrations and epigenetic clock measurements back to them. Newsletters and website updates are also to provide study updates and promote participant engagement.

### Strengths

One strength of the study includes measuring the baseline serum PFAS concentrations of approximately 2000 firefighters, leading to a better understanding of the impact of occupational activity history on PFAS. In addition, given that all interventions were requested by fire service leaders, there is more interest and determination to complete study activities. This study will also contribute to research into epigenetic age acceleration and potential interventions to improve it.

### Limitations

Our study has some limitations; first, there are significant barriers to plasma donation, including time commitment and distance, which can impede participant adherence. The Australian study being replicated identified an adherence rate of 93.7% to their interventions [11]. All the intervention arms require a time commitment; therefore, compliance is a concern [35]. Finally, not meeting enrollment targets will reduce our study's power to evaluate relationships between the intervention and PFAS levels and epigenetic age acceleration.

### Conclusions

The design, enrollment, and research activities of this randomized controlled trial provide an opportunity to observe PFAS interventions in real time and assess their feasibility and benefit.

## Appendix

Multimedia Appendix 1 Study consent form.

Multimedia Appendix 2 SPIRIT Checklist.

## Abbreviations

CPET: Cardiopulmonary Exercise Test
FCRP: Firefighter Collaborative Research Project
FFCCS: Fire Fighter Cancer Cohort Study
IRB: institutional review board
PFAS: per- and poly-fluoroalkyl substances
PFOA: perfluorooctanoic acid
PFOS: perfluoro octane sulfonate

## Data Availability

The datasets generated or analyzed during this study are not publicly available due to participant confidentially, and final datasets are accessible by approved study staff.
