# Supplementary material for: Interventions to Reduce Serum Per- and Poly-Fluoroalkyl Substances Levels, Improve Cardiovascular Risk Profiles, and Improve Epigenetic Age Acceleration in US Firefighters: Protocol for Randomized Controlled Trial
Source: JMIR Res Protoc. 2025 Apr 16;14:e67120. doi: 10.2196/67120 (PMC12044307; doi:10.2196/67120)
Supplement: Multimedia Appendix 1 [file resprot_v14i1e67120_app1.docx]

**University of Arizona**

**Consent to Participate in Research**

| **Study Title:** Firefighter Collaborative Research Project |
| --- |
| **Principal Investigator:** Jefferey Burgess, MS, MD, MPH |
| **Sponsor:** Arizona Board of Regents |

**Summary of the research**

**This is a consent form for participation in a research study.** Your participation in this research study is voluntary. It contains important information about this study and what to expect if you decide to participate. Please consider the information carefully. Feel free to ask questions before making your decision whether to participate.

The purpose of this research is to test interventions to reduce serum PFAS levels, firefighter cardiovascular risk, cognitive disease risks, and epigenetic age. Epigenetics is the study of how your genetic information is expressed differently based on your behaviors and environment. Epigenetic age is the measure of the rate of aging of your cells.

This study will attempt to determine how effective different interventions are at reducing health risks linked to per- and polyfluoroalkyl substances (PFAS) exposure, and other exposures to a variety of chemicals. Per- and polyfluoroalkyl substances (PFAS) are man-made chemicals that are commonly referred to as ‘forever chemicals’ due to their inability to be easily broken down in the human body and the environment. Previous research has shown that Arizona firefighters have higher PFAS levels in their blood than the general population.

If you agree to participate in this research, you will be enrolled in the study through the end of the study period in 2026. During this time, you will be asked to provide information about your relevant medical history, occupational and exposure history, food frequency and diet, physical activity, intervention progress and adherence, and biological collections. Your screening results will determine if you have high serum PFAS levels or low serum PFAS levels. The study arms that you can potentially be randomly assigned to will vary depending on if you have high or low serum PFAS levels.

If you have high serum PFAS levels, you will be preferentially enrolled into the blood donation arm, plasma donation, or control group for blood and plasma donation. The control group will not donate blood or plasma. Participation in the blood donation, plasma donation, and the blood and plasma donation control group will last for 12 months. Although you will be preferentially assigned to those groups, you may be randomized into any arm in the study, including zone 2 training and intermittent fasting, that you are eligible for.

If you have low serum PFAS levels, you may be enrolled in a cardiovascular fitness training program known as zone 2 training for 4 months, an intermittent fasting regime for 4 months, or be part of a control group that receives no intervention. Those with high PFAS levels not preferentially assigned to blood donation, plasma donation, or the control for blood and plasma donation may also be randomized into zone 2 training or intermittent fasting.

Participation is voluntary, and you may discontinue participation at any time.

Risks may include common risks associated with blood draws, blood donation, plasma donation, moderate exercise, or a change in your eating habits depending on the intervention and group you are randomly assigned to. You may not benefit directly from participating in this study.

**Why is this study being done?**

This study is being done to learn more about interventions that may reduce epigenetic age and cardiovascular risk, as well as overall health risks, associated with, but not limited to PFAS exposure. We hope to answer questions like:

- What interventions may be effective at addressing high blood PFAS levels?
- How do blood PFAS levels change from the beginning to end of the study?
- How effective are the interventions at lowering blood PFAS levels?
- How do the interventions affect cardiovascular, epigenetic age, and overall health risk?

**What will happen if I take part in this study?**

***All participants:***

This study is expected to last for three years. If you choose to take part this study, we will ask you to do these steps:

**Planned Surveys: Baseline and Follow-Ups**

Upon completion of this consent form, you will be asked to complete a baseline survey about your firefighting and work history, medical history, diet, exercise, and exposures. The survey should take you about 30 to 45 minutes. Study staff will use information provided in your baseline survey from the Fire Fighter Cancer Cohort Study to answer questions in the Firefighter Collaborative Research Project baseline survey.

We will ask you to complete additional follow-up surveys about your progress during your assigned intervention and ensure that you are completing assigned activities. Depending on the intervention, the number and frequency of the surveys may vary. You will also be asked to fill out a survey upon completion of the study.

You will receive links via text message and email to complete study surveys. Answers are entered into a secure electronic database, and you may end a survey at any time. Your answers to all survey questions should be accurately answered to the best of your understanding. Your answers to survey questions will be kept confidential. Your employer and/or your firefighting union will not have access to your individual answers or study results.

**Mindcrowd Cognitive Assessment**

We will also ask you to complete a cognitive assessment using a service called Mindcrowd. You will be provided with a link that is specific to you to fill out the assessment. You will have to consent to participate in Mindcrowd as it is a separate study from this study. You will not have to provide your name or any contact information to Mindcrowd. Mindcrowd will provide your results to you and send them to our study team.

**Biological Sample Collection**

Based on the serum PFAS concentrations measured in your screening blood sample, you will be placed in one of two groups: a group for those with high PFAS levels, or a group for those with low PFAS levels.

We will collect urine before you begin your intervention.

Throughout your intervention, we will collect blood samples to measure changes in PFAS levels, epigenetic age, and other exposures and outcomes. Biological collections locations will be chosen to accommodate participants, departments and regional locations. Blood will be collected by a trained phlebotomist, nurse, or paramedic.

Upon completion of the intervention, we will collect a final urine and blood sample to measure your post-treatment epigenetic age acceleration, change in serum PFAS levels, and other exposure and health outcomes.

***Blood and Plasma Donation for those with high serum PFAS levels (≥5 ng/mL PFOS):***

You may be preferentially randomly assigned into one of three intervention groups (like flipping a coin): blood donation, plasma donation, or a control group. The control group will not participate in any blood or plasma donation events. If you are assigned to either the blood or plasma donation group, you will be asked to donate at assigned frequencies over a 12-month period. We will collect an additional biological sample 4 to 6 months after beginning the intervention, in addition to the baseline and post-intervention samples if you are at a department in Arizona. You will also be asked to self-report on donations along with frequency of donation.

While it is preferred for participants with high serum PFAS levels to be randomly assigned to blood donation, plasma donation, or the blood and plasma donation control, these participants may be randomized into any arm of the study that they are eligible for. This means that if you are not placed into a group to donate blood or plasma, then you may be randomly assigned to zone 2 training, intermittent fasting, or their respective control groups if you are eligible.

***Zone 2 Physical Activity (Arizona only)***

If you are at a department in Arizona, you may be randomly assigned into one of the following groups: zone 2 training or a control group that will not participate in an intervention. You will participate in that treatment over a 4-month period.

For those participating in zone 2 training: The intervention will consist of 2 CPET tests: One at the beginning to assess your individual zone 2 heart rate range along with a follow-up test after 4 months of zone 2 training to evaluate changes. A Cardiopulmonary Exercise Test (CPET) is a non-invasive test designed to measure the performance of your heart and lungs at rest and during exercise. CPET involves exercising on an upright stationary bike while breathing through a mask and wearing external sensors. The testing will be carried out by Heartfit For Duty or 1582. You will be required to travel to and from the labs performing the CPET testing. We will ask you to self-report the results of your CPET test to study staff.

Zone 2 training is defined here as aerobic, cardiovascular training at 60% of one’s maximum heart rate. You will participate in zone 2 training for a minimum of 45 minutes per day, for a minimum of 4 days per week over the span of 4 months. You may be asked to complete additional follow-up surveys throughout the 4-month period. You will be allowed to do other workouts, in addition to the zone 2 exercises, but it must be after the zone 2 training if additional workouts are being done the same day. We will provide you with a wrist-worn heart and health tracker. We will ask that you wear the tracker at all times unless it is charging.

For the physical activity control group: You will be provided with a wrist-worn heart and health tracker, to be worn at all times unless it is charging.

Data from the wrist-worn tracker will be collected through a service called Fitabase and provided to our study team through Fitabase. Your tracker will be assigned an ID number that only those on our study team will be able to link back to you. Fitabase will only know you by the ID number assigned to your tracker. Fitabase will not permanently store your information and will not store any information after the study ends.

***Intermittent Fasting (Arizona only)***

If you are at a department in Arizona, you may be randomly assigned into one of the following groups: intermittent fasting or a control group that will not participate in an intervention. You will participate in that treatment over a 4-month period.

For those participating in intermittent fasting: you will be asked to participate in intermittent fasting for a minimum of 4 days per week over a span of 4 months. Intermittent fasting is defined here as having a 14-16 hour fasting period (no caloric intake for 14-16 continuous hours – coffee, black tea, and other clear drinks with no calories will be permitted) and an 8-10 hour eating period within a 24 hour day. You may be asked to complete additional follow-up surveys throughout the 4-month period.

For those in the intermittent fasting control group: you will be asked to complete follow-up surveys throughout the 4-month period. For all control groups: you may be asked for additional follow-up surveys.

I allow this study to take information from the baseline survey of the Fire Fighter Cancer Cohort Study to be used to answer the same questions in this study’s baseline survey.

Yes

No

I allow study staff to take information from surveys for this study following the conclusion of the study and share it with the Fire Fighter Cancer Cohort Study for my study records.

Yes

No

I allow this study to take information from a blood draw and urine samples collected in the Fire Fighter Cancer Cohort Study within the last 3 months and utilize them for baseline information for this study.

Yes

No

**How long will I be in the study?**If you agree to participate in this study, the length of your participation will range depending on the intervention group and arm you are randomized into. The study will end in 2026. This study is voluntary, and you are allowed to withdraw at any time.

If you are in the high serum PFAS intervention group and preferentially randomized into the blood or plasma donation treatments, you will be asked to donate blood and plasma for 12-months.

If you are randomized into the zone 2 training, zone 2 control, intermittent fasting, or intermittent fasting control, you will be asked to participate in these activities for 4 months.

You will be asked to complete additional surveys via text and REDCap throughout your participation in the study depending on what arm of the study you are randomized into.

**How many people will take part in this study?**

There will be approximately 1500 firefighters in this study.

# Can I stop being in the study?

You do not have to take part in this research study. Your choice to be in the study is **voluntary**. If you change your mind about taking part, you can leave at any time. Your choice to be in this study or not or to leave early will not affect your employment status or any benefits you are entitled to. If you withdraw from the study, the study team will stop collecting your information. The data that have already been collected may continue to be used once you withdraw from the study. Your collected samples will remain stored at the University of Arizona. The study team will discard them upon request. We may remove you from the study if you are non-compliant with study activities.

If you decide to withdraw your permission, you should contact the study team in writing.

Contact: Dr. Jeff Burgess
Phone: 520-848-4091
Email: COPH-FCRP-Study@arizona.edu

**What risks or benefits can I expect from being in the study?**

*Benefits:*

The data collected may not have an immediate benefit to you. However, your participation can help the study team identify avenues of possible interventions to reduce exposure levels, epigenetic age, cardiovascular risk, and overall health risk among firefighters.

If participating in blood donation, plasma donation, or the blood and plasma donation control group, you will receive your PFAS levels from the end of study blood draw. All participants who complete the study activities for their intervention or control group will receive the results of their epigenetic age. You may use these results to help inform your health or lifestyle choices. If participating in the zone 2 intervention or control group, you will receive the CPET results from pre-intervention and post-intervention.

We will also publish what we learn in medical journals, firefighter health and safety journals, public health journals, or environmental research journals.

*Risks:*

We expect the risks for being in this study to be minimal. Getting blood drawn may cause minor pain when the needle is inserted, bruising, or infection. There are no known risks with self-collection of urine samples. Donating blood or plasma may cause physical weakness or discomfort, pain, swelling, bruising, faintness, dizziness, nausea, a blown vein, and bleeding at the site of needle insertion. These risks are not specific to blood or plasma donation within this study.

Doing the CPET and zone 2 trainings poses risks associated with mild to moderate sustained physical activity. Common risks of the CPET and zone 2 include physical weakness, light headedness, and elevated heart rate. If you have previously had a heart problem, there is a small chance you may have a heart problem during the CPET. You will be closely monitored throughout the CPET to minimize risk and have the test discontinued if testing staff decides it is not safe for you to complete the test. The risks posed to you during zone 2 training depend on your existing level of physical fitness.

Wearing a wrist-worn heart and health tracker may cause slight skin irritation of the skin underneath the watch. These risks are not specific to wearing a wrist-worn heart and health tracker for this study.

Intermittent fasting poses physical risks of dehydration, nausea, fatigue, faintness, and hunger if you do not drink enough non-calorie containing fluids while fasting. Intermittent fasting also poses psychological risks including developing issues related to eating disorders.

Although all information on you, your health, samples you provide, and possible results will be kept in a secured database, there is a risk that an unauthorized person could get access to this data. We believe the chance this will happen is very small and we will do everything we can to protect your privacy. The other risk to you is the risk of breach of confidential information. We have several procedures in place to limit this risk and protect the confidentiality of your data. The existence of these procedures is mandated by law, as described below.

We will protect your privacy by labeling your samples and survey information only with a code and keeping the key to the code in a password-protected database.

There may also be other risks that we currently don’t know about.

During the study, we may find more information that could be important to you. This includes information that might cause you to change your mind about being in the study. If there are significant new findings that may impact your participation, you will be informed as soon as possible.

Your department liaison will know that you are participating in this study to assist our study team with scheduling follow up visits. However, identifiable study data, including survey responses and information about your health, will not be shared with your department’s liaison.

# Will I be paid for participating in the study or experience any costs?

You will receive no compensation from the research group for taking part in this study. The only cost to you is your time, as well as transportation to and from intervention and testing locations.

**What happens if I am injured because I took part in this study?**

Side effects (injury) can happen in any research study. These effects may not be your fault or the fault of the researcher involved. Known side effects have been described in this consent form. However, side effects that are not currently known may happen and require care. If you experience an injury or adverse event, please call Dr. Burgess at (520) 626-4918 immediately.

Compensation for pain, expenses from traveling to and from treatment locations, lost wages, and other damages caused by injury in the research activities of this study are not available.

This, however, does not waive your rights in the event of negligence. If you suffer an injury from participating in this study, you should seek treatment.

# Will my study-related information be kept confidential?

We are committed to respecting your privacy and keeping your personal information confidential. The University of Arizona has rules to protect information about you. Federal and State laws also protect your privacy. We have several procedures in place to protect the confidentiality of your data.

Generally, only people on the research team will know that you are in the research study and will be able to access any personally identifiable information about you such as name, residence, or contact numbers. Physical copies of such identifying information (including this consent form) will be filed in an official, secured area within a locked file cabinet; computer files will be protected with a password. Information that we collect about your identity, residence, and contact numbers will be kept separate from your medical information, and those data will only be available to research study personnel.

Your biological samples and medical information will be linked and identified solely by a unique study code, to protect your privacy. When information is shared with collaborators or submitted for publication, only coded information and summary information is shared.

It is anticipated that there will be circumstances where your study related information will be released to persons and organizations described in this form. Your information may be shared or disclosed with others to conduct the study, for regulatory purposes, and to help ensure that the study has been done correctly. These other groups may include:

- Office for Human Research Protections, Department of Health and Human Services, or other federal, state, or international regulatory agencies
- Arizona Board of Regents (ABOR)
- The University of Arizona (UA) and the UA Institutional Review Board
- Arizona State University and the ASU Institutional Review Board
- Heartfit For Duty for CPET
- 1582 for CPET
- Other study approved facilities that administer CPET testing

This research is covered by a Certificate of Confidentiality from the National Institutes of Health. This means that the researchers cannot release or use information, documents, or samples that may identify you in any action or suit unless you say it is okay. They also cannot provide them as evidence unless you have agreed. This protection includes federal, state, or local civil, criminal, administrative, legislative, or other proceedings. An example would be a court subpoena.

There are some important things that you need to know. The Certificate DOES NOT stop reporting that federal, state, or local laws require. Some examples are laws that require reporting of child or elder abuse, some communicable diseases, and threats to harm yourself or others. The Certificate CANNOT BE USED to stop a sponsoring United States federal or state government agency from checking records or evaluating programs. The Certificate DOES NOT stop disclosures required by the federal Food and Drug Administration (FDA). The Certificate also DOES NOT prevent your information from being used for other research if allowed by federal regulations.

Researchers may release information about you when you say it is okay. For example, you may give them permission to release information to insurers, medical providers or any other persons not connected with the research.

**Will my specimens be sold for commercial profits?**

Your specimens will not directly be sold for commercial profit. The information/specimens may

be used in this research or other research and shared with other organizations as noted. You

will not share in any commercial value or other compensation from products developed using

the information/specimens.

**Will I hear back on any results that directly impact me?**

If participating in blood donation, plasma donation, or blood and plasma donation control group, you will receive your PFAS levels from the end of study blood draw. All participants who complete the study activities for their intervention or control group will receive the results of their epigenetic age. If participating in the zone 2 intervention or control group, you will receive the CPET results from pre-intervention and post-intervention.

**Will Whole Genome Sequencing be done with my specimen?**

No Whole Genome Sequencing is planned for any specimens collected.

**Will my study-related information be used for future research?**

Information that may identify you may be used for future research or shared with another researcher for future research studies without additional consent. Information collected about you and biospecimens collected from you will be used for this research and may also be used for other research studies. We may also share information and specimens with other institutions for research. Before using the information and specimens for other research, the study team will remove information that identifies you so the individuals performing the research will not know who the information and specimens came from.

# Who can answer my questions about the study?

If at any time you feel you have had a research-related injury, or for questions, concerns, or complaints about the study you may contact **Jeff Burgess, MD, MS, MPH** at **520-848-4091** or via email at COPH-FCRP-Study@arizona.edu.

For questions about your rights as a participant in this study or to discuss other study-related concerns or complaints with someone who is not part of the research team, you may contact the University of Arizona Human Subjects Protection Program Director at 520-626-8630 or online at <https://research.arizona.edu/compliance/human-subjects-protection-program>.

# Signing the consent form

I have read (or someone has read to me) this form, and I am aware that I am being asked to participate in a research study. I have had the opportunity to ask questions and have had them answered to my satisfaction. I voluntarily agree to participate in this study.

I am not giving up any legal rights by signing this form. I will be given a copy of this form**.**

|  |  |  |  |  |
| --- | --- | --- | --- | --- |
| **Printed name of subject (First, Last)** |  | **Signature of subject** |  | **Date** |
| **Street Address** |  |  |  |  |
| **City, State, Zip Code** |  |  |  |  |
| **Phone Number** |  |  |  |  |
| **Optional Phone Number** |  | **E-mail address** |  |  |
|  |  | **Repeat E-mail address** |  |  |

______________________________________________________________________________

STUDY STAFF ONLY

Name of person obtaining consent: ______________________________________

Signature of person obtaining consent: ______________________________________

Institution of person obtaining consent: ______________________________________

Date: ______________________________________
